# Measurement of TBM Disc Cutter Wear Using Eddy-Current Sensor in Different TBM Chamber Conditions: Insights from Laboratory Tests

**DOI:** 10.3390/s25072045

**Published:** 2025-03-25

**Authors:** Minsung Park, Minseok Ju, Jungjoo Kim, Hoyoung Jeong

**Affiliations:** 1 Department of Energy Resources Engineering, Pukyong National University, Busan 48513, Republic of Koreaamine77@pukyong.ac.kr (M.J.); 2 Power System Research Laboratory, KEPCO Research Institute, KEPCO, Daejeon 34056, Republic of Korea; jungjoo.kim@kepco.co.kr

**Keywords:** eddy-current sensor, disc cutter, wear, measurement environment

## Abstract

The TBM disc cutter, which is the main cutting tool of tunnel boring machines (TBMs), is replaced when it is excessively worn during the boring process. Disc cutters are usually monitored by workers at cutterhead chambers, and they check the status and wear of disc cutters. Manual measurement occasionally results in inaccurate measurement results. In order to overcome these limitations, real-time disc cutter monitoring techniques have been developed with different types of sensors. This study evaluates the distance measurement performance of an eddy-current sensor for measuring disc cutter wear via a series of laboratory experiments. This study focused on identifying the effects of various measurement environments on the sensor’s accuracy. The study considered conditions that the eddy-current sensor may encounter in shield TBM chambers, including air, water, slurry, and excavated muck. Experiments were conducted using both a small-scale disc cutter and a 17-inch full-scale disc cutter. The results indicate that the eddy-current sensor can accurately measure the distance to the disc cutter within a specific range and that its performance remains unaffected by different measurement environments.

## 1. Introduction

In tunnel boring machines (TBMs), the disc cutters installed on the cutterhead are the main components of mechanical excavators to excavate rock formations [1]. The disc cutters experience wear and damage during continuous excavation processes. Excessive wear and breakage of the disc cutter reduce the cutting efficiency of cutting tools and increase the TBM’s downtime. Therefore, it is important to monitor the status of disc cutters at appropriate times. Empirical prediction methods, such as different wear index tests and rock properties like quartz content [2,3,4], are commonly employed to estimate the wear rate and consumption of disc cutters in the design stage. However, these methods are limited to estimating the required number of cutters roughly and assuming that only normal wear patterns occur. Therefore, these methods have limitations in being applicable to cutter replacement management, as they do not encompass the various wear and damage patterns exhibited by disc cutters in real conditions [5]. For these reasons, cutter replacement schedules are usually determined based on manual inspections by workers. This process involves workers entering the cutterhead chamber with specialized tools or guides to visually measure cutter wear, and it allows them to identify damaged cutters that should be replaced. However, the inside of the TBM chamber is consistently vulnerable to the inflow of soil and groundwater, with a high potential for exposure to hazardous conditions like high pressure. To address this issue, workers operating within the chamber undergo specialized training to adapt to these circumstances, and they should perform the tasks while wearing heavy safety equipment (diving suits, oxygen devices, etc.). Nevertheless, it must be recognized that the risk of personal accidents is consistently present during operations.

In order to overcome the various limitations related to the manual inspection of disc cutters, several previous studies have utilized various types of sensors to measure and monitor the condition of disc cutters [6,7,8].

Guo et al. (2013) [6] introduced a cutter wear measurement system using an ultrasonic sensor to measure the amount of wear on TBM disc cutters. They highlighted that the working conditions of ultrasonic sensors (i.e., the frequency of transducers, excitation voltage, and amplification signal) are crucial in the measurement system. The ultrasonic sensors offer advantages of intuitive measurement principles and straightforward measurements in preferable working conditions. However, there are some limitations to be considered in cutter wear measurement. The ultrasonic waves emitted and transmitted by the ultrasonic sensor have the characteristic of being reflected by nearly all surfaces. This characteristic implies that the distance measurement of the ultrasonic sensor can be influenced by the excavation materials inside the cutterhead chamber, such as soil, rock debris, gravel, and other materials, during TBM excavation. Moreover, the speed of ultrasonic wave propagation and the extent of penetration or reflection depend on the properties of the medium such as density, bulk modulus, and acoustic impedance. Given these characteristics, ultrasonic waves can travel through various media from the transmitter to the receiver, potentially causing fluctuations in their propagation speed by the time they return. Furthermore, it has been observed that the measurement performance of ultrasonic waves is affected by the temperature of the medium through which they travel.

In Gong et al. (2021) [8], the application of magnetic sensors was utilized to investigate the wear of disc cutters. In this study, a magneto-resistive sensor designed with a Wheatstone bridge circuit structure was employed. There are four magneto-resistors within the bridge arm, and due to their sensitivity to magnetic fields, the output voltage changes in response to variations in the magnetic field. The correction curves for wear values were obtained through a series of laboratory tests in four different media: air, water, slurry, and muck. As a result, it was observed that even in the presence of different media, there was minimal variation in wear measurement values when using magneto-resistive sensors. Therefore, the measurement values of magneto-resistive sensors were reported to remain unaffected by non-magnetic materials (such as excavated soil or rocks) located in the vicinity of the sensor. Based on the site application, it was concluded that the wear measurement system utilizing magnetic sensors also exhibited accurate measurement results. However, as reported in that study, the output values of the magnetic sensor (e.g., voltage, current) exhibited a nonlinear relationship with the amount of disc cutter wear, indicating the necessity of a correction function to accurately map the sensor output to the wear amount. Additionally, field measurement results showed significant fluctuations, highlighting the need for additional data filtering to improve measurement reliability.

On the other hand, eddy-current sensors have been applied to measure the wear of disc cutters in previous studies [7,9,10]. The eddy-current sensor is well known for its high-precision distance measurement performance and stability, making it a preferred choice in various mechanical engineering fields. Eddy-current sensors have many practical applications in industry, including position sensing, non-destructive testing, and in induction motors [11,12]. Additionally, previous studies have been extensively conducted to enhance the measurement performance of eddy-current sensors in various measurement environments [13,14]. As a result of these efforts, many sensor manufacturers provide high-performance commercialized eddy-current sensors. Lan et al. (2019) [7] introduced a wear measurement system for disc cutters and presented a case study of its application in the Yinsong Tunnel in China. They presented a series of laboratory experiments conducted to calibrate the measurement values obtained from the eddy-current sensor. Particularly, in scenarios where geological materials such as rocks or soil are situated between the sensor and the disc cutter, which are similar to the cutting conditions of the disc cutter, they emphasized the significance of adjusting the measurement values to consider their influence and handle measurement anomalies. The calibrated measurement system was validated for accuracy through linear cutting tests performed while cutting rock formations before its application on-site. The accuracy of the measurement system was verified in the process of cutting rocks, and the measurement error of the eddy-current sensor was reported to be at the level of 0.15 mm. The measurement system, following calibration procedures and accuracy validation in the laboratory, was subsequently tested in practical application on a TBM excavating the Yinsong Tunnel in China. The diameter of the TBM is 7.95 m (Gripper TBM), and a total of 51 disc cutters (sensor cutters: 17 inches, face/gauge cutters: 19 inches) were installed. Among them, the sensors were placed on disc cutters 29 and 37, which are positioned relatively towards the outer edge of the face cutters. When comparing real-time measured wear with actual values obtained through manual inspections, discrepancies were reported, with the maximum error ranging from 0.43 to 0.93 mm across eight measurement instances. This indicated that the eddy-current sensor performed relatively well in measuring cutter wear in real time.

Based on the results of previous studies, the eddy-current sensor has been evaluated as having significant potential for real-time measurement of TBM disc cutter wear. However, to enable its application to various types of TBMs (e.g., EPB and slurry), an assessment of its applicability in different chamber environments such as excavated soil, water, slurry, and air is necessary. Therefore, this study focuses on evaluating the accuracy of TBM disc cutter wear measurements using the eddy-current sensor in various chamber conditions. Additionally, experiments were conducted to examine measurement conditions that may affect the sensor’s accuracy, with the aim of proposing a set of optimal conditions.

## 2. Materials and Methods

### 2.1. Eddy-Current Sensor

#### 2.1.1. Principles of an Eddy-Current Sensor

**Figure 1:**
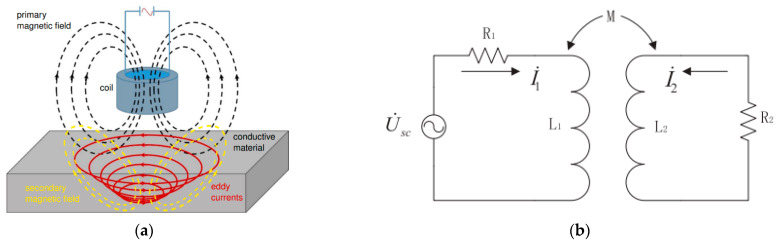
The working principle of the eddy-current sensor: (**a**) magnetic interaction between the eddy-current sensor and target [15], (**b**) the magnetic field circuit between the eddy-current sensor and target.

The eddy-current sensor, which operates based on electromagnetic induction, is a sensor that utilizes changes in coil inductance caused by eddy currents generated within a conductor. Figure 1a shows a schematic representation of the electromagnetic phenomenon occurring in the vicinity of the eddy-current sensor.

The alternating current flowing within the coil generates a high-frequency alternating magnetic field (primary magnetic field) around the coil, which induces eddy currents on the surface layer of the conductor. These eddy currents, in turn, create a secondary magnetic field within the surface layer of the conductor, with a direction opposite to that of the primary magnetic field. The principle of the eddy-current sensor involves measuring the changes in coil parameters and inductance resulting from the interaction of these magnetic fields. When representing this process with an electrical circuit composed of an alternating power supply, coil and conductor resistances, and mutual inductance, it can be depicted as shown in Figure 1b, where R_1_ represents the resistance of the coil, L_1_ denotes the coil’s inductance, R_2_ stands for the equivalent resistance of the measured object, L_2_ signifies the equivalent inductance of the measured object, and M represents the mutual inductance between the coil and the measured object. Inductance represents the ratio of the counter-electromotive force generated by electromagnetic induction due to changes in current flowing through a circuit. The coefficient of mutual inductance (M) is denoted as $K\sqrt{L_{1}\times L_{2}}$. The coefficient (K) represents the distance between the coil and the conductor, and as the distance increases, (K) tends to converge to 0, leading to the mutual inductance (M) also converging to 0. Conversely, as the distance between the coil and the conductor decreases, the coefficient (K) tends to converge to 1, resulting in a change in the mutual inductance (M) to $\sqrt{L_{1}\times L_{2}}$. Measuring such changes in mutual inductance forms the fundamental principle of distance measurement using eddy-current sensors. Additionally, eddy currents exhibit strong penetration capabilities in non-ferrous ores like rocks, enabling the utilization of magnetic field energy between the energy coil and the conductor (disc cutter) to measure factors such as the conductor’s position, surface morphology, and thickness.

#### 2.1.2. Structure of an Eddy-Current Sensor

**Figure 2:**
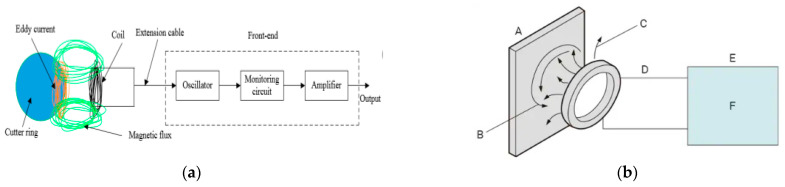
The general structure of an eddy-current sensor. (**a**) Eddy-current sensor structure [16]. (**b**) Structure of eddy-current displacement sensor (this study).

The structure of the eddy-current sensor consists of a coil, extension cable, and front-end, as illustrated in Figure 2a. The internal detection circuitry within the front-end consists of an excitation circuit, a detector, and an amplifier circuit. It is isolated from the external environment to prevent interference with external signals. Figure 2b provides a simple schematic drawing of the eddy-current-type digital displacement sensor used in this study (A: target object, B: eddy current, C: high-frequency magnetic field, D: coil, E: amplifier, F: high-frequency excitation circuit). Eddy-current sensors use variations in excitation state based on changes in impedance to measure distances, using the aforementioned structure.

#### 2.1.3. Selection of an Eddy-Current Sensor

**Figure 3:**
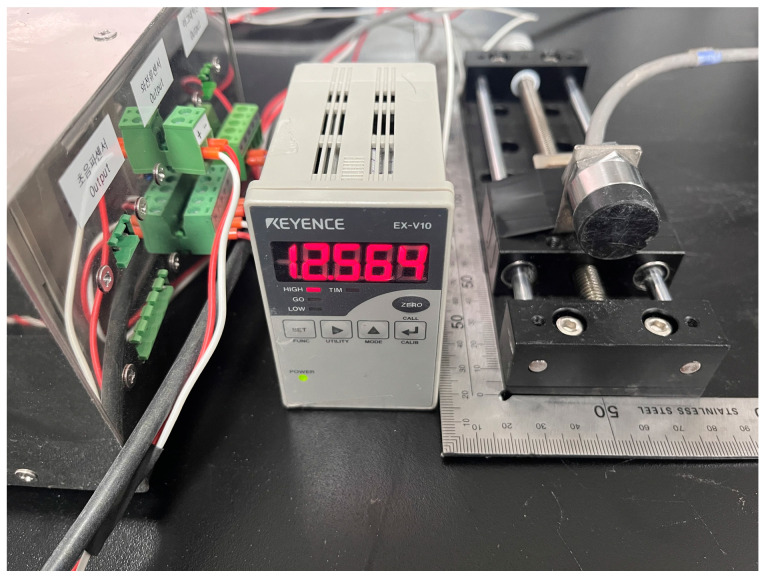
A photo of the eddy-current sensor and other components used in this study.

Eddy-current sensors are categorized into two types: detection-type sensors, which determine the presence of the target object (material) within a measurable range, and displacement sensors, which measure the distance between the sensor and the target object. In order to measure the wear of the disc cutter, the latter type of eddy-current sensor should be considered. In this study, the specifications of eddy-current sensors for distance measurement provided by several sensor manufacturers were reviewed to select the proper model of eddy-current sensor. Considering the environment of the TBM cutterhead, the sensor should be compact with a small diameter, possess waterproof capabilities to withstand chamber pressure and water and slurry infiltration, and be capable of measuring distances within an appropriate range. Upon reviewing the specifications of commercially available eddy-current displacement sensors, we selected the EX-422V (sensor head) and EX-V10 (amplifier unit) models provided by Keyence Corporation. Table 1 summarized the specification of the sensor. The sensor can offer a maximum measurement range of 10 mm (typical for ferrous materials, guaranteed value) and a resolution of approximately 2 μm, while the weight of the sensor head is 200 g. Figure 3 displays a photo of the eddy-current sensor system, including the sensor unit, amplifier unit, and digital indicator.

### 2.2. Experimental Procedures

#### 2.2.1. Preliminary Test for Small Discs

In this study, the experimental program was divided into two categories. First, we carried out the preliminary test for small diameters of disc cutters to evaluate the measurement accuracy of eddy-current sensors in laboratory conditions. The main purposes of the preliminary test are (1) checking the test procedure of eddy-current sensors for distance measurement, (2) confirming the ability of the sensor to measure the distance between the sensor and the disc cutter, and (3) identifying the relationship between output and preset distance.

**Figure 4:**
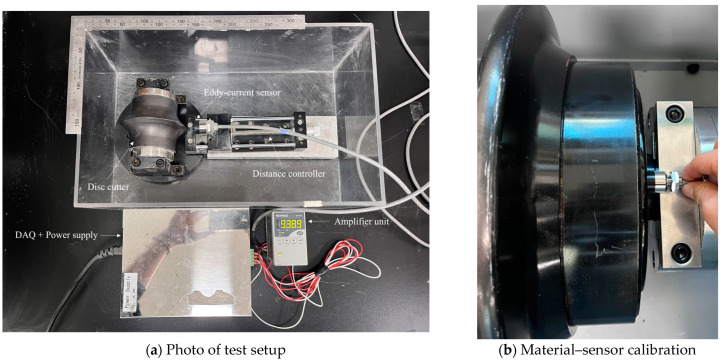
Preliminary test using eddy-current sensor.

**Figure 5:**
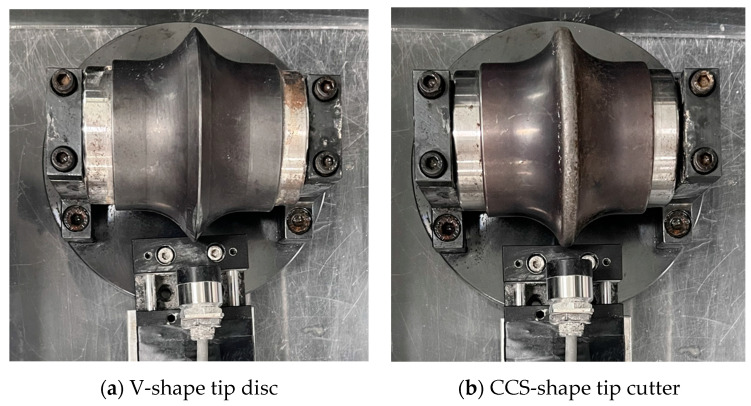
Photos of small-size disc cutter with different shapes of tips.

The testing equipment for the preliminary test was prepared, and it consisted of an eddy-current sensor, amplifier unit, moving table with ruler, small disc cutter, and DAQ. The experimental setup is illustrated in Figure 4a. The distance measurement test using the eddy-current sensor was conducted as follows. First, a calibration process was performed for the target material (the same material used for small and 17-inch discs). This process involved matching the output voltage to the corresponding distances within the desired measurement range. Through this calibration, the output voltage was recorded at two reference positions: when the sensor was in contact with the material surface (defined as 0 mm) and at the maximum measurable distance. Using the distance and output data from these two points, the eddy-current sensor was able to determine the measurement distance for any arbitrary position based on the linearity between distance and output voltage. In the laboratory conditions, the wear of the disc cutter was simulated by the distance between the fixed disc cutter and the movable sensor. The small disc cutter was fixed in the test box, while the sensor on the moving table could be moved by rotating a lever for the desirable distance. In addition, a small-scale disc cutter was used for the test. The disc cutter had a diameter of 100 mm, and the tip width was divided into two types: a V-shape with a 2 mm tip and a CCS (constant cross section)-shape with a 7 mm tip (Figure 5). The reason for the distinction of the tip shape is that the measurement accuracy of the eddy-current sensor can be affected by the surface shape of the target object. If the measurement surface is too narrow relative to the sensor head’s diameter, the interaction with the magnetic field induced by the eddy currents generated on the surface may be distorted. Therefore, this study assessed the effect of the disc cutter tip width on the measurement results before proceeding with further experiments.

#### 2.2.2. Test for 17-Inch Discs

**Figure 6:**
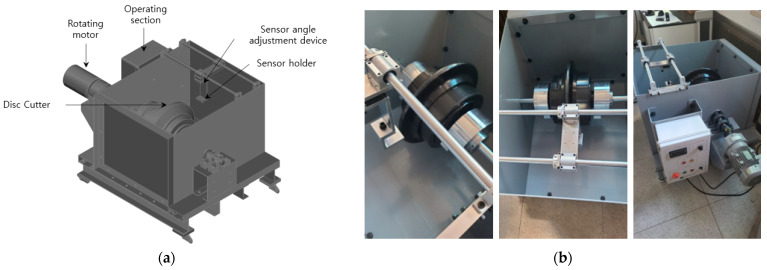
Test device for measuring the wear of a 17-inch disc cutter with rotation within different media: (**a**) 3D overview diagram and (**b**) photos of individual components.

After a series of preliminary tests, the same procedures were planned for a 17-inch disc cutter to investigate the effect of measurement conditions on the accuracy of eddy-current sensors in real scale. The specialized test apparatus was manufactured for the real-scale test. Figure 6a illustrates the configuration and a schematic 3D diagram of the test apparatus, while Figure 6b presents photographs of the test equipment. The developed test apparatus is capable of accommodating a full-scale 17-inch disc cutter, with adjustable rotational speed. Considering the typical RPM of a tunnel boring machine (TBM) and the diameter of the disc cutter, the rotational speed can be controlled up to a maximum of 100 RPM. Additionally, a dedicated mounting unit was fabricated to install the sensor for measuring the distance to the disc cutter. This sensor mounting unit was designed to allow adjustments in both the vertical and horizontal directions, enabling precise control of the measurement distance and orientation.

#### 2.2.3. Measurement Environments

**Figure 7:**
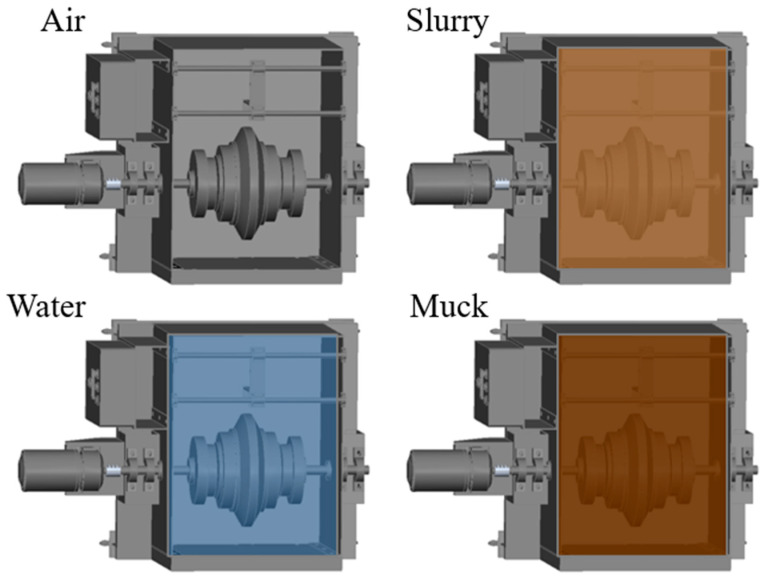
A conceptual diagram illustrating the different chamber environments (air, water, slurry, and muck) simulated in the test device.

In addition, as wear measurement sensors perform measurements within the TBM chamber, they should be able to perform accurate measurements under various conditions encountered within the TBM chamber, including underwater, slurry, and air environments (Figure 7). The slurry environment was prepared by mixing bentonite with water at a concentration of 7.5%, considering the typical concentration used in slurry (or mix-shield) TBMs, while the muck environment was prepared using excavated material from an EPB-type TBM tunneling site. The excavated material consists of soil and rock debris, making it suitable for simulating the actual conditions inside the TBM chamber. Therefore, in this study, to examine the influence of the surrounding medium on the measurements, the eddy-current sensor and the measuring conductor were placed in underwater, bentonite slurry, and excavated muck environments for both distance measurement tests in two scales. It should be noted that the eddy-current sensor employed in the experiment is designed with waterproof and pressure-resistant features.

## 3. Results

### 3.1. Preliminary Test for Small Disc Cutter

#### 3.1.1. Accuracy in Distance Measurement

**Figure 8:**
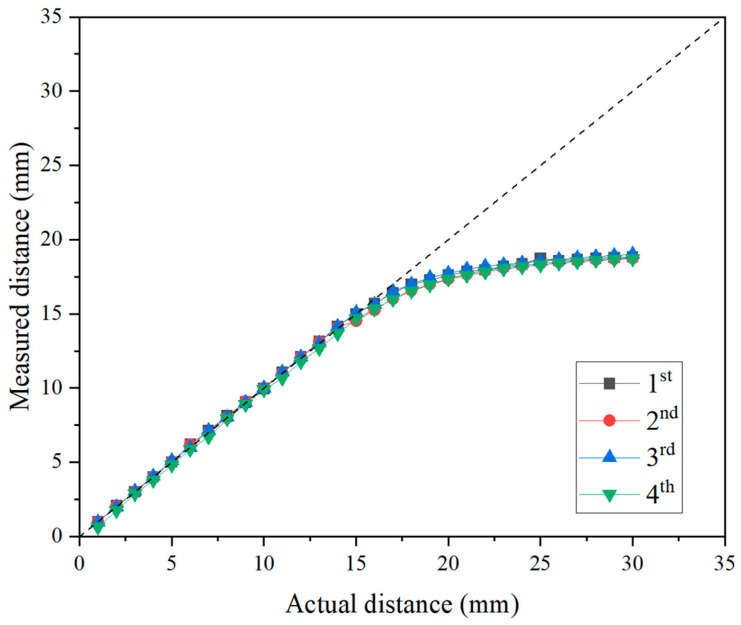
Results of distance measurement test using eddy-current sensor in air conditions.

To assess the distance measurement performance of the eddy-current sensor, a distance measurement experiment was conducted by placing a small disc cutter and the eddy-current sensor in an air environment. Figure 8 presents the results obtained from four repeated tests conducted under the specified conditions (a dotted line indicates 1:1 scale). The distance was measured up to 30 mm with 1 mm intervals. It was concluded that a linear measurement trend was observed up to a distance of approximately 16 mm. The manufacturer’s specified measurement limit refers to the threshold that satisfies both linearity, where the actual distance and measured value exhibit a linear relationship, and precision simultaneously. However, when the distance exceeds 16 mm, the measured values exhibit a nonlinear increasing trend rather than a linear trend. This nonlinearity of eddy-current sensor measurement has also been reported in previous studies [7,16]. This nonlinearity in eddy-current sensors is inherently based on the fundamental measurement principles of the sensor itself. The nonlinearity of eddy-current sensors primarily arises from the distance-dependent attenuation and distribution of eddy currents, which affect the sensor’s output signal. As the measurement distance increases, the induced magnetic field spreads, reducing eddy-current density and causing a nonlinear decline in signal strength. Additionally, the relationship between the magnetic field strength and distance follows an inverse square law, leading to a nonlinear response at extended ranges. Variations in sensor coil characteristics, including changes in the induced magnetic field shape, further contribute to nonlinearity. Moreover, the electrical conductivity and magnetic permeability of the measured material influence eddy-current dissipation, exacerbating nonlinear behavior, particularly in non-ferrous metals and alloys. To compensate for this nonlinearity, various correction methods, including multi-segment linear approximation, correction functions, and machine learning-based algorithms, have been proposed in previous studies [17,18,19]. Therefore, in order to utilize eddy-current sensors within a broad measurement range where nonlinear segments are present, it is deemed necessary to derive proprietary calibration coefficients or functional expressions.

**Figure 9:**
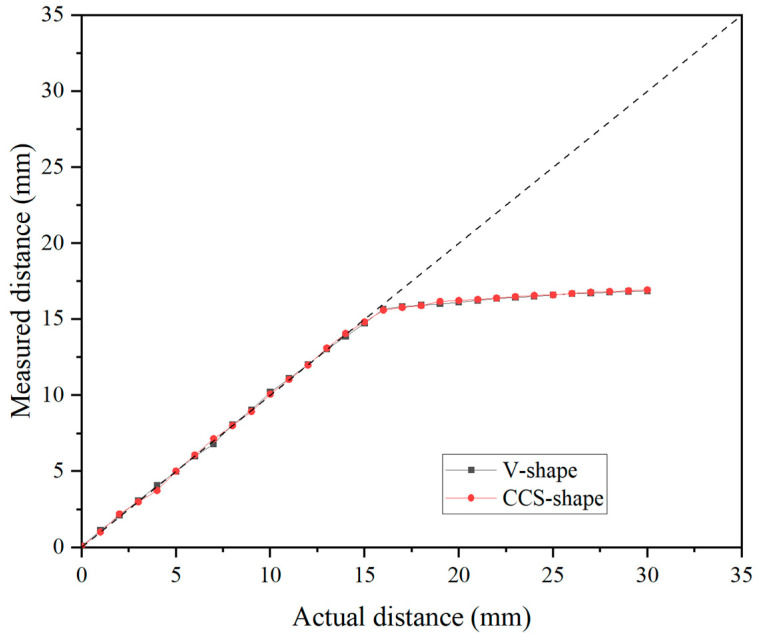
Results of distance measurement test using eddy-current sensor for different cutter tips.

Furthermore, Figure 9 shows the effect of tip width on the results of the distance measurement. The results indicated that the accuracy of the eddy-current sensor was not affected by the tip width. The two sets of results were completely consistent with each other, exhibiting the same trend even in the region where nonlinearity appeared beyond 16 mm. Therefore, this study concluded that the tip width of the disc cutter does not affect the measurement accuracy of the eddy-current sensor. In addition, it can be concluded that the measurement performance is not affected and the eddy-current sensor can provide stable measurement values even if the cutter tip width of the disc cutter changes due to wear in the actual measurement environment in the TBM site.

#### 3.1.2. Effect of Measurement Media

**Figure 10:**
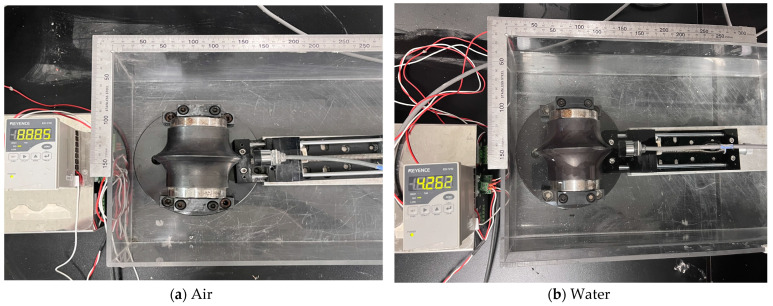

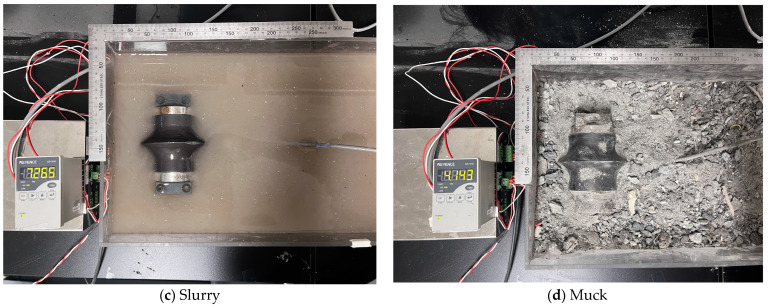
Preparation of eddy-current sensor test for small-size discs in different environments.

As mentioned earlier, we considered four different environments, namely air, water, slurry, and muck. In order to simulate the different measurement conditions, the test box was filled with different media, as shown in Figure 10. The slurry was prepared with a concentration of 7.5%, considering the real operational environment of the shield TBM. For the excavated soil conditions, actual excavated soil retrieved from a TBM construction site was used. As demonstrated by the results of the previous tests conducted in an air environment, the tip width of the disc cutter did not affect the measurement results. Therefore, the tests were conducted using the disc cutter with the narrower tip width (V-shape), as it was expected to present a more challenging measurement environment.

**Figure 11:**
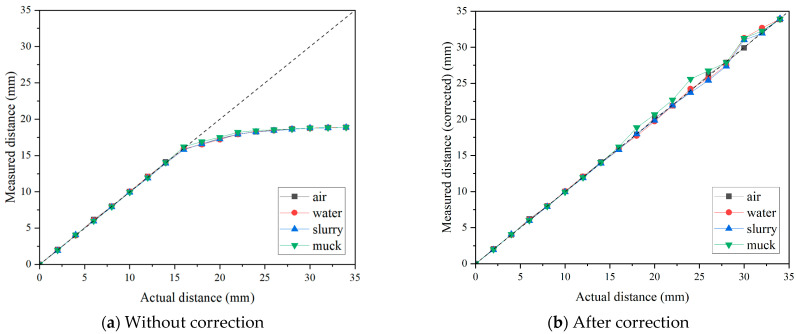
Effect of the measurement environment on the results of distance measurement for small size disc with the eddy-current sensor.

Figure 11a presents the distance measurement results for the eddy-current sensor in each considered environment. The results were highly consistent with those obtained in the previous tests described in Figure 7 and Figure 8. Up to 16 mm, the measured values precisely matched the actual values, but beyond this point, the measurements exhibited a nonlinear increase rather than a linear trend. In the muck condition, the measured values showed fluctuation compared to the other conditions before reaching 16 mm, which was attributed to the difficulty in precisely controlling the sensor’s distance in this condition. This can be considered as an experimental error. In order to correct the values in nonlinear ranges (i.e., above 16 mm), correction functions were derived based on the linear regression models. Table 2 summarizes the correction functions and corresponding results for each environment. It should be noted that the correction functions were derived based on the results obtained under air conditions. Considering the principles of eddy-current sensors and the simplicity of the correction process, nonlinearity above 16 mm was approximately corrected with several linear functions within discrete ranges, because eddy-current sensors fundamentally assume linearity between reference points. The correction results are presented in Figure 11b, and the results indicated that the eddy-current sensor demonstrated good accuracy across all measurement ranges, regardless of the measurement environment. Sensor manufacturers do not allow users to apply arbitrary corrections through a pre-amplifier beyond the initial calibration process described earlier. However, by utilizing the internal correction options commonly provided in a separate DAQ system, users can input the correction functions to obtain accurate measurements in nonlinear ranges. Based on these findings, it was confirmed that the distance measurement performance of the eddy-current sensor is not affected by the presence of a medium other than air between the sensor and the disc cutter. This result is attributed to the measurement principle of eddy-current sensors, which rely on detecting changes in coil inductance induced by the conducting material. However, this also implies that the presence of other magnetic materials in the TBM chamber may lead to measurement errors.

### 3.2. Seventeen-Inch Disc Cutters

#### 3.2.1. Effect of Measurement Media

**Figure 12:**
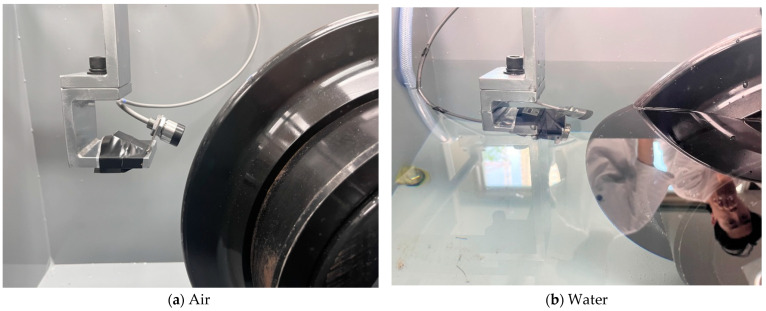

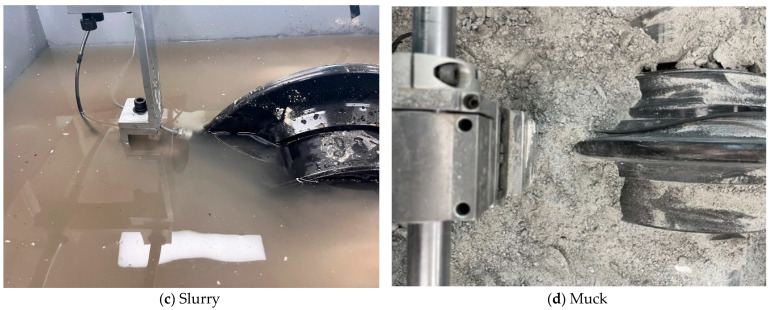
Preparation of eddy-current sensor test for 17-inch disc cutter in different environments.

After a series of preliminary tests for small-size disc cutters, additional tests were carried out for 17-inch disc cutters to investigate the effect of measurement conditions on the accuracy of eddy-current sensors. Therefore, in this study, we aimed to validate the eddy-current sensor technique for full-scale disc cutters and to analyze the strengths and weaknesses of the sensor. To achieve this, a 17-inch disc cutter was installed and a device capable of adjusting the rotation speed was utilized as described in Figure 6. Following the same procedure as that of the test for small disc cutters, we simulated four different environments, namely air, water, slurry, and muck, inside of the test equipment, as shown in Figure 12.

**Figure 13:**
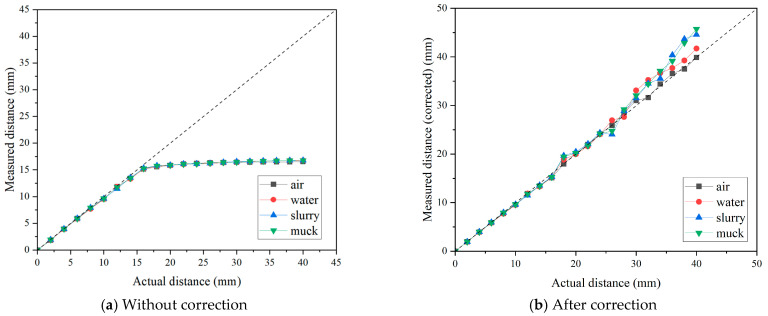
Effect of the measurement environment on the results of distance measurement for 17-inch disc by the eddy-current sensor.

Figure 13a presents the distance measurement results for 17-inch disc cutter in each considered environment. The results for the 17-inch disc cutter showed no significant deviation from those of the preliminary laboratory experiments. The distance measurement test was carried out up to 40 mm with 2 mm of intervals, and as observed in previous laboratory experiments, the results exhibited a linear trend up to 16 mm, after which a nonlinear interval appeared. This consistent trend also highlights the necessity of deriving an appropriate correction function to accurately translate measurement values in the nonlinear interval into actual distances. Therefore, following the same process as the former analysis of the small disc cutter, correction functions were derived to calibrate the nonlinear ranges. Multiple discrete linear functions were obtained by selecting segments with similar linear slopes, and regression equations were derived using the results from the air conditions. The correction results are presented in Figure 13b, while Table 3 summarizes the correction functions and corresponding results for each environment. As a result, the measurement accuracy remained stable across all measurement environments, achieving approximately 95% accuracy on average. As the measurement distance increases, the measurement error gradually increases. This issue can be mitigated by reducing the interval for deriving the correction functions, thereby improving the accuracy of measurements in the nonlinear range. Furthermore, the measurement results within the four different environments (i.e., air, water, slurry, and muck) showed no significant differences. In conclusion, consistent with the findings from the preliminary tests, the eddy-current sensor’s performance in measuring disc cutter wear is not affected by the surrounding medium.

#### 3.2.2. Effect of Measurement Angles

The above test results correspond to the desired test condition where the sensor’s measurement direction is precisely perpendicular to the surface of the disc cutter. As previously explained, the eddy-current sensor measures the distance to the disc cutter by detecting the eddy currents generated on the surface of the ferromagnetic material and the corresponding changes in magnetic force. Therefore, in the case of objects with irregular surface geometries, such as a disc cutter, the measurement values of the eddy-current sensor may be affected by the measurement direction.

**Figure 14:**
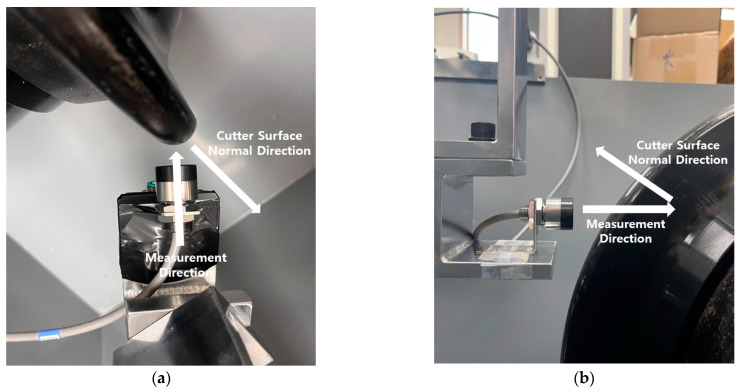
Definition of two types of measurement angles according to the position of the eddy-current sensor: (**a**) indicates the measurement angle according to the rotation around the vertical axis with a fixed vertical position, and (**b**) indicates the measurement angle according to the vertical position of the sensor.

In this study, additional tests were carried out to assess the effect of variations IN measurement directions (angles) on the measurement accuracy of the eddy-current sensor. This could provide useful information in determining the optimal installation position of the wear sensor for disc cutters. The angle between the sensor and the disc cutter can be categorized into two scenarios: (1) the measurement angle according to the rotation around the vertical axis with a fixed vertical position, and (2) the measurement angle according to the vertical position of the sensor as defined in Figure 14.

**Figure 15:**
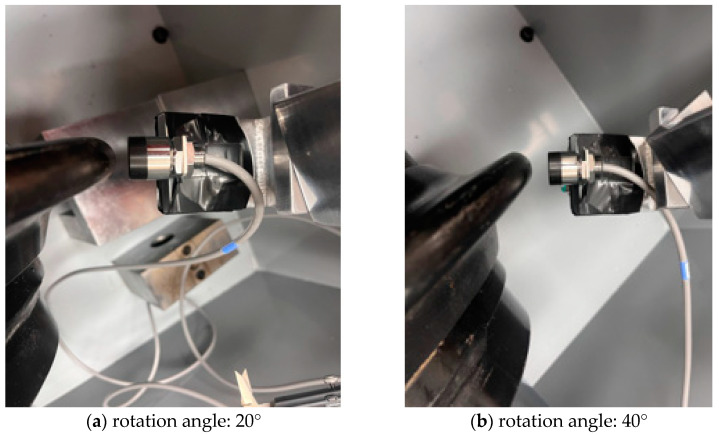
Setting of rotation angles between disc cutter and sensor.

**Figure 16:**
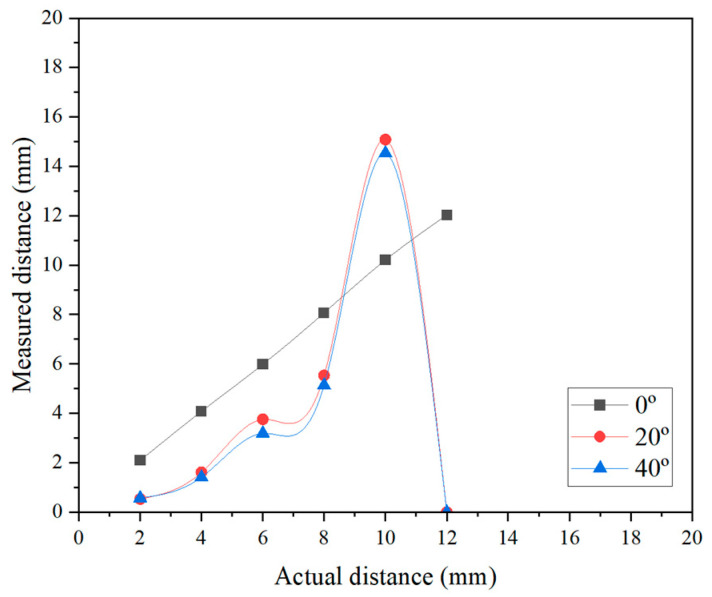
Example of distance measurement results from disc cutter according to measurement angle of eddy-current sensor.

The distance measurement tests were performed to examine the effect of the rotation angle on the accuracy of the eddy-current sensor. The rotation angles were considered to be 20° and 40° compared to the 0° case (Figure 15). The actual distance between the sensor and the disc cutter was measured using a digital caliper, and ranged from 0 mm to 12 mm with 2 mm intervals. The corresponding variations in measurement values are presented in Figure 16. The results for the 20° and 40° angles indicated a significant deviation from the actual values, and did not exhibit a linear trend compared with that of the 0° angle. Additionally, the measurement range appeared to be greatly reduced. In the case of the 20° angle, a nonlinear increase was observed from 7 mm distance. Moreover, beyond 11 mm distance, the eddy-current sensor showed an “FFFF” (error) signal. In the case of the 40° angle, similar results were obtained to those in the 20° case. In contrast, for the measurement angle defined under Scenario 2, the results indicated that when a slight angular deviation of within 5 degrees occurred, accurate measurement was not achieved. The results demonstrated that when the installation angle of the eddy-current sensor deviated from the optimal measurement orientation (i.e., the normal direction to the disc’s surface), significant discrepancies showed between the measured values and the actual distances. The results are supported by a recent study [17] that investigated similar measurement conditions and observed comparable results. They reported that as the angle between the sensor head and the measurement surface increases from 5° to 11°, the measurement accuracy continuously decreases, and the linearity in this range also significantly deteriorates. In conclusion, it is essential to install the eddy-current sensor in a manner that ensures measurements are conducted precisely perpendicular to the surface of the disc cutter.

## 4. Conclusions

In this study, the performance of an eddy-current sensor, utilized for measuring disc cutter wear, was evaluated through a series of laboratory experiments. The primary purpose of this study was to investigate the impact of different measurement environments on the distance measurement accuracy of the eddy-current sensor. Specifically, this study considered the environmental conditions the sensor may encounter when used as a wear measurement tool for disc cutters, including air, water, slurry, and excavated muck. Experiments were conducted using both a small-scale disc cutter and a 17-inch full-scale disc cutter, revealing that the eddy-current sensor employed in this study provided linear measurement values up to 16 mm of wear. Beyond 16 mm, nonlinear measurement values were observed, indicating the necessity for additional calibration to ensure accurate measurements in this range. Therefore, this study addressed measurement inaccuracies in the nonlinear range by deriving multiple linear correction functions. The application of these correction functions enabled distance measurements of the disc cutter with an accuracy of 95% up to 40 mm. Given that the wear limit of a disc cutter is 25 mm, these results suggest that the eddy-current sensor has the potential for effective application in real-time wear monitoring of disc cutters. It is noteworthy that the findings of this study are based on undamaged disc cutters rather than including various wear patterns, such as normal wear, uneven wear, or damaged cutters, commonly observed in the field. Future research should focus on evaluating the measurement performance of the sensor on disc cutters with diverse wear conditions obtained from actual TBM operations. Furthermore, the influence of the sensor’s measurement angle on distance accuracy was experimentally evaluated. The results demonstrated that for precise measurements, the eddy-current sensor must be aligned perpendicular to the surface of the disc cutter. Any deviation in the measurement angle leads to inaccuracies, highlighting the sensitivity of the sensor’s orientation to measurement precision. The results of this study were obtained from a new disc cutter. A limitation of this study is that it utilized new, undamaged disc cutters rather than incorporating various wear patterns, such as normal wear, uneven wear, or damaged cutters, commonly observed in the field. Future research should focus on evaluating the measurement performance of the sensor on disc cutters with diverse wear conditions obtained from actual TBM operations. However, since the findings of this study were obtained under controlled laboratory conditions, further investigation is required before applying eddy-current sensors to measure disc cutter wear in actual TBM operations. Additional environmental factors such as the high pressure and elevated temperature within the cutterhead chamber may influence the measurement accuracy and performance of the eddy-current sensor. Therefore, future research should focus on these aspects to ensure the sensor’s reliability and effectiveness in real TBM applications.

## Figures and Tables

**Table 1:** The main specifications of the eddy-current sensor used in this study.

Measurement Range	Sampling Speed	Measurement Precision	Sensor Head Diameter	Weight	Waterproof Grade
10 mm	15,000 num/s	2 μm	Ȼ 22 × 35 mm	200 g	IP67

**Table 2:** The correction functions for measurement with the eddy-current sensor (small disc).

Range (mm)	Correction Functions (y = a + bx)	Accuracy (%)			
		Air	Water	Slurry	Muck
0–16	y = x	99.2	98.6	98.4	97.5
16–22	y = −31.457 + 2.977x				
22–28	y = −164.19 + 10.297x				
28–34	y = −583.77 + 32.729x				

**Table 3:** The correction functions for measurement with the eddy-current sensor (17-inch disc).

Range (mm)	Correction Functions (y = a + bx)	Accuracy (%)			
		Air	Water	Slurry	Muck
0–16	y = x	98.1	95.9	95.0	94.9
16–22	y = −99.318 + 7.532x				
22–26	y = −253.37 + 17.101x				
26–32	y = −775.95 + 49.101x				
32–40	y = −1660.73 + 102.891x				

## Data Availability

Data are contained within the article.
